# Food taboos, health beliefs, and gender: understanding household food choice and nutrition in rural Tajikistan

**DOI:** 10.1186/s41043-019-0170-8

**Published:** 2019-08-07

**Authors:** Katharine McNamara, Elizabeth Wood

**Affiliations:** Department of Environmental & Global Health College of Public Health and Health Professions, University of Florida, 1225 Center Drive, P.O. Box 100182, Gainesville, FL 32610-0182 USA

**Keywords:** Food taboos, Health beliefs, Gender, Nutrition, Health

## Abstract

Household nutrition is influenced by interactions between food security and local knowledge negotiated along multiple axes of power. Such processes are situated within political and economic systems from which structural inequalities are reproduced at local, national, and global scales. Health beliefs and food taboos are two manifestations that emerge within these processes that may contribute beneficial, benign, or detrimental health outcomes. This study explores the social dimensions of food taboos and health beliefs in rural Khatlon province, Tajikistan and their potential impact on household-level nutrition. Our analysis considers the current and historical and political context of Tajikistan, with particular attention directed towards evolving gender roles in the wake of mass out-migration of men from 1990 to the present. Considering the patrilieneal, patrilocal social system typical to Khatlon, focus group discussions were conducted with the primary decision-making groups of the household: in-married women, mothers-in-law, and men. During focus groups, participants discussed age- and gender-differentiated taboos that call for avoidance of several foods central to the Tajik diet during sensitive periods in the life cycle when micronutrient and energy requirements peak: infancy and early childhood (under 2 years of age), pregnancy, and lactation. Participants described dynamic and complex processes of knowledge sharing and food practices that challenge essentialist depictions of local knowledges. Our findings are useful for exploring entaglements of gender and health that play out across multiple spatial and temporal scales. While this study is situated in the context of nutrition and agriculture extension, we hope researchers and practitioners of diverse epistemologies will draw connections to diverse areas of inquiry and applications.

## Background

The recognition of good nutrition as a fundamental driver for sustained social, economic, and political development has led to global efforts to eradicate malnutrition [1]. These efforts have aided in reducing the prevalence of malnutrition worldwide; however, accessing safe, nutritious food in adequate quantities continues to be a struggle for approximately 815 million people across a variety of contexts, regardless of the GDP of their nation [2]. Spatial and temporal patterns of food distribution are heterogeneous, causing disproportionate levels of malnutrition among some groups of people [3]. Intersectional approaches to malnutrition can aid in considering the combined, complex interactions between health and the mutually constituting subjectivities that contribute to vulnerability: gender, age, ethnicity, and caste, among others [4]. In this article, we consider *vulnerable* groups those whose intersecting subjectivities convey greater susceptibility to malnutrition and severity of its effects (e.g., diarrhea, stunting, wasting), and face the greatest risk of long-term health consequences due to poor nutrition. We simultaneously explore the overlap of vulnerability *and* privilege as critical for engaging diverse agents of change who reflect a multitude of health experiences [5].

Malnutrition takes a variety of forms and is often expressed comorbidly alongside other health conditions. According to Soeters et al., malnutrition is “a subacute or chronic state of nutrition in which a combination of varying degrees of over- or undernutrition and inflammatory activity have led to a change in body composition and diminished function” ([6], p. 708). While the definition proposed by Soeters et al. guides our conceptualization of malnutrition, this article places particular emphasis on the implications of *undernutrtion* due to its pervasiveness within the study site: Khatlon Province, Tajikistan.

Tajikistan faces the highest rate of undernutrition in Central Asia with approximately 5% of children under the age of 5 years suffering from acute undernutrition (wasting), 30% from chronic undernutrition (stunting), and 11% from underweight [7]. Accessibility and availability of food is most concerning in rural areas of the country, where food insecurity is concentrated [7]. Khatlon province, a largely rural region located in southwest Tajikistan, is highly vulnerable to malnutrition due to the interaction of poverty, tough agroecological conditions, and high rates of male migration for work (38%) [8]. These factors are complicated by gender hegemonies, wherein gender expectations are performed, intertwine with, and are perpetuated within the broader socioecological system that dominate subaltern masculinities and femininities [4, 9]. Ultimately, the dynamic between power, social systems, and complex food landscapes influences *how much* of *what kind* of food is consumed and *by who*.

Since the 1970s, development practice has largely targeted the immediate drivers of malnutrition through nutrition-specific interventions like micro- and macronutrient supplementation [10]. However, a growing body of research demonstrates that nutrition-specific programs are not sufficient to reach global targets as they fail to address the complex socioecological determinants of malnutrition relevant at multiple scales of intervention [11, 12]. In response, nutrition research and practice increasingly emphasize the importance of the *underlying* determinants of malnutrition through nutrition-sensitive interventions [13]. Within the agriculture sector, such programs seek to influence the availability, accessibility, and diversity of food [12]. The agriculture extension system (AES) is considered particularly well-positioned to execute nutrition-sensitive efforts because of its close engagement with communities and families and potential to bridge multiple pathways to improved nutrition through local agro-food systems [11, 14].

Tajikistan faces the challenge of developing effective strategies for nutrition-sensitive agriculture amidst a dearth of literature relevant to its geographic and cultural context. However, in combination with cases from Uzbekistan, a neighboring country that shares some sociocultural similarities and shared history with Tajikistan as a former Soviet state, this small body of work can help scholars and practitioners glean relevant points of entry into more comprehensive nutrition interventions. For example, in both Uzbekistan and Tajikistan, it is common for elderly parents to live in the home of their youngest son and his family in a multigenerational household [15, 16]. Complex relations emerge within in this familial arrangement that are central to the decisions made around food production, food preparation, and diet. While the power dynamics in this context are diverse, the interaction of age and gender often situate young women and children at the low-end of intrahousehold hierarchies [17]. Relations between senior and junior women (e.g., between mother-in-law and daughter-in-law), widowed mother and son, husband and wife, and junior and senior men (e.g., between father and son) fluidly maintain a matrix of interacting hierarchal structures [15]. Relationships between mothers-in-law and daughters-in-law are particularly important to decision-making around food and strongly influence household nutrition [17].

Tajikistan has experienced continual demographic changes  since the late 1990s, spurred by growing rates of male out-migration. Today, approximately 40% of the working-age population leave the country to pursue work abroad; The majority of migrating workers are men from rural areas [16]. Naturally, demographic transformations are accompanied by changes in gender relations and expectations at multiple levels of society. While gender in any context is multifaceted, encompassing a range of discursive and performative processes by which masculinities and femininities are (re)constructed (for example through labor and specialized knowledge) [18], rapid and ongoing changes to national and local demographics contribute additional complexity to local gender relations. Despite the need for flexible research methods to capture these interwoven interactions, categorical gender analysis—which interprets men and women as static groups—remains widespread in the health literature. Nowhere is this more apparent than in work that equates *gender* with *women* and the fundamental linkages between men’s and women’s health are overlooked (see [19]). Ultimately, such approaches risk framing health and gender as “women’s issues” and essentializing men and women as either the perpetrators or victims of hierarchical dynamics, respectively, despite variation within these fluid subjectivities [4]. A gender relations approach that is responsive to complex and changing interactions “not only within but across identities and analytic categories” ([4], p. 1676), is therefore crucial for understanding gender and health in the context of Tajikistan.

The research presented in this article builds on the findings of a previous investigation that explored how Tajik intrahousehold dynamics affect the allocation of food resources and, ultimately, nutrition (see [17]. At the request of local agriculture extensionists, we framed our “initial dive” into food-related decision-making practices with the purpose of identifying recommendations to target malnutrition through AES. Entering this investigation, we expected to observe similar patterns to those documented in other contexts with prominent malnutrition and similar intrahousehold hierarchies. However, we found that food taboos and health beliefs shaped intrahousehold dietary practices in unexpected ways—a pattern not reflected by other studies in Central Asia.

In the early-to-mid 1900s, early Anthropological endeavors on the subject of taboos conceptualized such practices as irrational, pseudo-science avoidances "which, in their simplest forms, are almost as instinctive as those of the lower animals" ([20], p. 14). Later, taboos were reinterpreted as instrumental, rational practices that regulate complex social systems [21]. Over the last decade, the trajectory of scholarly approaches has evolved towards complex, integrated visions wherein the socio-ecological functions of taboos are entangled with symbolism and spirituality, history and politics, and economic and environmental conditions [22]. In this article, we apply insights from contemporary inquiry into taboos (see Meyer-Rochow 2009, Golden and Comaroff 2015) in tandem with theoretical contributions from anthropology, geography, and masculinity studies to call attention to the specific ways that Tajik food taboos shape and *are shaped by* gendered experiences and knowledges around health.

According to Meyer-Rochow (2009), the word food taboo is used to describe the deliberate avoidance of a specific food item "for reasons other than simple dislike from food preferences" ([23], p. 2). In some cases, food taboos protect from health hazards [24], in others they facilitate environmental conservation or safeguard limited resources [22, 25]. Thus, intimate connections between food taboos and social-ecological systems punctuate cultural practice [17]. Food taboos can indicate specialized knowledge of specific household members and the responsibilities and roles attached to certain subjectivities. In this way, both awareness and practice of taboos may be most aparent within sub-groups most involved in their preservation [18]. While food taboos are embedded within community health beliefs, the later reflects values associated with a given activity or practice. More specficially, health beliefs encompass a breadth of attitudes, perceptions, and values stemming from various sources of health-related knowledge. Another distinction lies in how health beliefs emerge and are preserved within a community. Taboos involve the co-evolution of practices within the fabric of social power structures. Health beliefs, in contrast, reflect diverse renderings of health concepts that may be important at both individual and group (e.g. household, community) levels; Thus, health beliefs are not necessarily tied to multigenerational knowledge-sharing. Health beliefs and food taboos are interconnected, however, within the unique social-ecological system of the context from which they emerge; For example, health beliefs may inform adaptive food restrictions. Finally, both concepts are flexible and respond to changes in environmental, political, and economic configurations [23].

As seen in other contexts, food taboos in Khatlon Province may reflect intrahousehold power dynamics along the axes of age and gender as social expectations performed through food practices. Building on the findings of our earlier work, we aim to explore how food security in Khatlon Province is mediated by taboos and health beliefs that govern dietary practices during critical points in the human life and along gendered subjectivities [25]. For example, young mothers and children experience increased nutrient and energy demands during pregnancy and lactation, and during the first 2 years of life, respectively. Thus, food restriction at these phases life can magnify the health impact of seasonal scarcity, crop failure, and other disturbances to the agro-food system on women and young children due to the interaction of social status and increased dietary requirements during “nutrient-expensive” stages of life [3]. Experiences of both women and men are crucial to understanding the determinants of household nutrition status. However, no regional study of household health has considered the position of men—much less their nutrition knowledge and practices—beyond their role as “head of household” or as the standard next to which women's health status is evaluted. In light of recent gendered demographic transformations and their role as a destabilizing force in the Tajik household [26], such considerations are necessary to capture the sociocultural nuances associated with diet and nutrition and the multiplicity of health effects incurred by all household members.

This article explores and characterizes the social dimmensions of food taboos and health beliefs in Khatlon Province and their potential impact on household nutrition by analyzing a subset of the data collected from the household decision-making study described above. We apply a gender relations approach by recognizing “gender dynamics and the circumstances under which they interact to influence health opportunities and constraints” ([9], p. 2); Analysis across gender categories is necessary to capture nuance within health and nutrition experiences. Our ultimate goal is to draw linkages between local knowledge and the evolving political, economic, and environmental context of Khatlon Province that came forward in the data as central to local adaptive strategies around health. We do this by presenting taboos as dynamic, flexible, and in a constant state of emergence in response to ongoing socioecological changes; the topics of shifting demographics, agricultural labor, and unspecific taboos are most salient in this respect. To our knowledge, no other studies have documented the critical role of food taboos and health beliefs in household nutrition and dietary practices in the Central Asian Region. By filling the void of locally relevant research on connections between gender dynamics and health, this study holds implications for nutrition-sensitive programs seeking to address the underlying causes of undernutrition.

## Methods

A team from the University of Florida (UF) conducted this study in February 2017 in collaboration with partners from the Tajikistan Agrarian University (TAU) and the Feed the Future Tajikistan Agriculture and Water Activity (TAWA) project. Prior to data collection and participant recruitment, permission to conduct this research was granted by the Institutional Review Board II (IRB II) at the University of Florida. UF principal investigators (PIs) were experienced in qualitative methods and had extensive background in global public health and nutrition. Research assistants from UF and TAU were invaluable members of the research team and worked alongside PIs from data collection to analysis. All research assistants from UF were in the master’s in public health (MPH) program and were recruited based on their previous involvement in public health research alongside the PIs and interest in conducting nutrition-related research abroad. TAU research assistants were recommended by TAU faculty based on the focus of their graduate studies in agriculture extension and communication and their familiarity with the rural, agrarian context of Khatlon province. UF PIs provided training in qualitative research methods to research assistants from both universities before fieldwork was conducted. UF research assistants received training on focus group discussion (FGD) and interview methods, effective probing questions, and real-time note-taking strategies. TAU research assistants were trained in the same competencies with the addition of real-time oral and written translation and word-for-word translation and transcription of recorded data. Together, PIs and trained research assistants met with agriculture extension agents from TAWA—this organization refers to extension agents as “Extension Home Economists” (EHEs), we will use this terminology from this point on—to deliver training on FGD facilitation and to develop a data collection strategy to implement during FGDs that would involve collaboration between EHEs, research assistants, and PIs. Due to the EHEs’ familiarity with participants through their extension work, it was decided that EHEs would lead the FGDs with community members while support roles were filled by TAU research assistants (responsible for translating in real-time to UF researchers and asking probing questions) and UF research assistants and PIs (responsible for managing recordings, note taking, and posing probing questions for translation to TAU research assistants).

Content analysis forms the theoretical approach of this study and was chosen deliberately for two reasons: (1) the dearth of existing literature and theories within the context of interest and (2) our ultimate goal of describing and characterizing a phenomenon, in this case the intrahousehold dynamics that govern food-choice and practices in Khatlon Province. The use of content analysis was crucial to our inductive approach to data analysis through which codes, categories, and themes were directly drawn from the data [27].

Prior to conducting FGDs with community members, four key informant interviews (KIIs) were conducted with nutrition and maternal and child health specialists from the World Health Organization, UNICEF, German Corporation for International Cooperation, and a local health clinic in Khatlon Province to provide researchers with information on household food and nutrition-related practices within the region. KIIs also allowed researchers to gain insight into best practices for nutrition-related field work in Tajikistan, specifically Khatlon Province. Participants were purposively selected based on the in-country partners’ knowledge of organizations working on nutrition in the region. Following KIIs, the FGD instrument was tested in Yavon, a village within Khatlon province, among mothers with children under 10 years of age. The instrument was revised and adjusted for cultural competency.

FGDs took place in 12 villages across five districts in Khatlon Province (Shahrtuz, Jomi, Khuroson, Sarband, and Vakhsh), which were selected due to their location within USAID’s Feed the Future Zone of Influence and connection to ongoing extension work with EHEs. In 2014, TAWA EHEs established women’s groups in collaboration with the Women Entrepreneurship for Empowerment Project (WEEP), which seeks to provide leadership and skill-building activities related to agriculture and nutrition to women of reproductive age. Through their work with the WEEP women’s groups, EHEs have built strong working relationships and trust within those communities, making EHEs ideal facilitators of these discussions. FGDs were conducted among three target populations: in-married women, mothers-in-law, and men. These participant groups were chosen based on the patrilineal and patrilocal social organization of Tajik households. We defined the participant groups according to their relationship with the in-married women due to her central role in diet-related decisions. “Men” refers to the husbands of the in-married women or males in the same age cohort as men of marrying age. “Mothers-in-law” refers to mothers of the in-married women’s husband, or mothers of men of marrying age. Due to household hierarchies, key informants strongly recommended separating these three groups during FGDs for honest responses and to ensure full participation of each group member in the discussion. Based on this recommendation, data from two FGDs was excluded from our analysis because the groups included both in-married women and mothers-in-law. In these two cases, EHEs were unable to separate the in-married women from their mothers-in-law without risking household conflict. Thirteen homogenous FGDs were analyzed for the purposes of this study: seven FGDs with in-married women, four with mothers-in-law, and two with men. FGDs varied in size (from 5 to 12 participants), with fewer total male participants as compared to women due to the high rate of male migration for work and their subsequent absence in many villages. Both the number of FGDs conducted with men and the number of male participants in each FGD clearly reflect these trends.

Each FGD was conducted by an EHE of matching gender to the participants with a TAU and UF research assistant present. During the discussion, a TAU research assistant translated the discussion in real-time while one UF research assistant transcribed verbatim using a laptop and a second UF research assistant took notes and asked probing questions via the facilitator. All FGDs were recorded to capture any lost data and were later reviewed and compared to the transcripts by a TAU research assistant to ensure data quality. Due to the stigma of illiteracy, oral consent was collected in the participants’ native language: Tajik, Uzbek, or Russian. Before initiating the discussion, TAU students or EHEs read the consent agreement aloud in the local language. The theoretical approach of this study was reflected in the structure and style of the focus groups, which were framed with open-ended questions relating to dietary practices and household decision-making around food. Targeted probing questions based on respondents’ comments allowed for a participant-directed discussion. When discussions surrounding specific infant and young child feeding (IYCF) practices arose in the FGDs, participants were asked to define the age at which those practices were exercised.

Transcripts from the 13 homogenous FGDs form the empirical basis of this study. Researchers and research assistants from UF carried out data analysis using the constant comparative method where coding and analysis take place simultaneously [28]. Intercoder reliability was strengthened by building consensus between coders through intensive group discussion to develop a coding framework. Analysis was organized using Excel, in which each code was defined concisely. Follow-up discussions between coders were continual throughout the data analysis process to continually check interpretive convergence. Once all data were coded using QSR International’s NVivo 11 software, segments of the transcripts were retrieved and consolidated into an Excel matrix organized by theme, subtheme, participant group, and interpretation. From this, researchers defined recurrent themes and patterns. *Food taboos* and *misconceptions* emerged as sub-categories nested within *determinants of food choice*. Following analysis, we recoded *misconceptions* as *health beliefs* to convey the legitimacy of local knowledge in shaping health practices. Due to the rich discussions by participants, researchers conducted an additional analysis of the data subset that related specifically to *food taboos* and *health beliefs*. This allowed researchers to develop a more nuanced understanding of food taboos and health beliefs as they relate to nutrition in Khatlon Province.

## Findings

The findings presented here build on our previous work on the intersections of household decision-making and nutrition. Our analysis targets a subset of that data relating specifically to food taboos and health beliefs, two themes that arose as critical determinants of household decision-making around food in the preceding work. Discussions around food taboos and health beliefs arose organically from an open-ended question: “Are there foods you avoid eating? Why?” This question was intentionally gender-neutral and probing questions, similarly, did not use gendered pronouns. Several themes and subthemes emerged that characterize food taboos in Khatlon Province according to *who* the taboo affects and *when.* There were also several health beliefs that followed similar patterns, affecting certain individuals during specific phases of the life cycle. Finally, a small portion of food taboos were found to be unspecific (uninfluenced by gender or age). The themes developed during analysis are presented according to a life-cycle approach: (1) food taboos during pregnancy, (2) health beliefs around breast-feeding, (3) food taboos during infancy and childhood, and (4) food taboos unspecific to gender or stage in life.

### Food taboos during pregnancy

Antenatal food taboos were pervasive across participant groups and villages. However, while men were aware of restrictive antenatal taboos, women (in-married women and mothers-in-law) provided reasoning to detail why those practices were necessary. From the perspective of in-married women and mothers-in-law, exclusion of certain foods was intended to protect and support maternal health. For example, a mother-in-law stated, “When they have morning sickness they cannot eat oily foods.” Restriction of oily foods is practiced early in pregnancy to reduce the likelihood and severity of morning sickness. However, one mother-in-law explained that intake of oily foods may be limited throughout pregnancy and that, in general, *“*pregnant women don’t eat as much oily food.”

FGDs across participant groups pointed to a general restriction of carbohydrate consumption during pregnancy. Men voiced their awareness of this practice by noting specific high-carbohydrate staple foods that are not consumed by pregnant women. The foods mentioned by men included *osh* (a rice dish) and *mantou* (dumplings). Women noted a more comprehensive list of avoided foods, adding noodles, bread, other baked goods. One mother-in-law summarized this list as “foods with carbohydrates”. When asked why carbohydrates are restricted, women explained that “if you eat these kinds of foods or meals you will have difficulty during birth” (In-married woman). Participants from women’s FGDs explained that consumption of carbohydrates during pregnancy leads to excessive weight-gain and a risky delivery because high gestational weight gain (GWG) “makes [the] baby very big” (mother-in law). Pistachios and nuts, a high-fat food item, were also restricted from the diet for the same reason.

These food taboos may have emerged recently in Khatlon Province due to their reported connection to recommendations from local physicians. Mothers-in-law explained that “[pregnant women] are told [by doctors] not to eat pistachios and nuts because they think the babies will be fat”. This observation was supported in the FGDs with in-married women, one of who stated, “Doctors tell [pregnant women] not to eat nuts, noodles, bread, foods rich with carbohydrates and recommend to eat more fruits and juice.” Both quotes suggest women consider restriction of certain foods key to physician recommendations for the prenatal diet. In-married women additionally emphasized the importance of fruits and vegetables to maternal diets during their focus groups.

### Health beliefs around breastfeeding

Breastfeeding practices only emerged as a topic of conversation within women’s FGDs. Within these discussions, women participants reflected on the challenges of breastfeeding amidst breastmilk insufficiency, financial hardship, and inadequate breastfeeding promotion, awareness, and education. Insufficient milk production was the most commonly cited reason for early termination of breastfeeding and appears to be a relatively common challenge among young mothers in the region. As a result of insufficient breastmilk, infants receive supplemental foods at an early age (before the age of 6 months). For example, one in-married stated, “my baby was four months old, and I gave him cow’s milk because I didn’t have enough milk.” While some participants reported that women may purchase formula when breastmilk is insufficient, there was an overall preference to supplement infant diets with animal-source milk. Women cited financial or nutritional grounds to support this choice. For many women and their families, formula is too expensive to consider as a breastmilk replacement. For others, animal milk is simply preferred due to the perception that it is more nutritious. As one in-married woman explained: “I am not in condition to buy formula, but I buy cow milk for my children which may be healthier.”

### Food taboos during infancy and childhood

Following pregnancy, food taboos prevalent during pregnancy decline alongside emerging food taboos specific to their new infant. Similar to antenatal taboos, food taboos during infancy and childhood are intended to protect children during vulnerable stages in life. Across all participant groups, infants and children were considered highly sensitive to gastroenteric upset based on the belief that they cannot digest foods as effectively as adults —prevention of upset stomach was the most common reason for excluding certain foods from infant’s and children’s diets.

Discussions around egg avoidance departed from the narrative of preventing gastric upset. Participant groups also diverged in their reasons for restricting egg consumption among young children. For example, mothers-in-law believed that “babies who didn’t start talking, they shouldn’t eat eggs, because it will influence, they will start speaking very late." In the previous quote, the mother-in-law highlights the importance of restricting egg consumption during a critical period for cognitive development. Since children usually begin speaking around 18–24 months, we estimated that this participant group considered children under the age of 2 years most at-risk to the perceived detrimental effects of eggs. Mothers-in-law also mentioned that “…if [children] eat eggs they have the problems with their stomach." This respondent connected egg taboos with the common motivation to protect children from gastric upset. Men, meanwhile, diverted from both of these reasons and believed that eggs should be excluded from children’s diets because they are high-calorie foods.

Across focus group discussions, participants differentiated between appropriate and taboo foods for young children between 6 months and 2 years of age according to two primary categories: light/ soft (considered appropriate) and heavy/hard foods (considered taboo). Light/soft and heavy/hard foods were grouped together in our analysis because some villages used the words “light” and “heavy” to describe appropriate and taboo foods, respectively, while “soft” and “hard” were used by others. The difference in categorization of foods between villages emerged due to linguistic variation between the study sites. Translation of Uzbek, Tajik, and Russian into English resulted in slightly different interpretations. However, we considered these words linguistic equivalents based on the parallel descriptions of each food type given by participants. Participants referred to soft-textured, mild-tasting, and carbohydrate-rich foods as light/soft. Hard/heavy foods were often diluted with water to make them more palatable for children between 6 months and 2 years of age. Participants described oily and carbohydrate-rich staple foods as hard/heavy. Foods within these categories included *fatir* (a type of bread), *sambusa* (a meat or vegetable filled pastry), *osh* (a rice dish), and *mantou* (a meat or vegetable-filled dumpling). Again, participants state these foods should be excluded from young children’s diets “because [it is] difficult to digest these meals” (In-married woman). One in-married women explained that hard/heavy foods can be introduced “starting at two, three years, but in very small amounts starting from two years” while another stated that, for some children, these dietary practices continue past the age of 2 years due to taste preference or household dietary practices.

Within women’s FGDs, in-married women and mothers-in-law believed some fruits and vegetables should be excluded from the diets of children under 2 years because of their association with gastric upset. This practice was affirmed by an in-married woman who stated, “We don’t give them tomato, cucumber, watermelon, and grapes, because of diarrhea.” Fruits and vegetables grown under certain conditions, inside greenhouses (according to in-married women) or with contaminated irrigation water (according to mothers-in-law), were considered more likely to cause gastric illness in young children. Participants identified accounts from neighboring villages and personal experiences with sick children as their sources of information. One in-married woman recounted, “Some people even died when they ate watermelons and melons, some people died from botulism,” a mother-in-law, meanwhile, voiced the general observation that children “[have diarrhea] after they eat cucumber.” Avoidance of fruits and vegetables in this case is protective of young children, who women in FGDs identify as most vulnerable to food-borne illness.

### Food taboos unspecific to gender or stage in life

Some food taboos and health beliefs were reported as unspecific to gender or stage in life. Instead, unspecific food avoidances applied to all members of the family. However, only in-married women and mothers-in-law discussed unspecific food taboos in their FGDs. Women identified imported food and “foods grown with chemicals” (mother-in-law) as unsafe for human consumption. Imported foods were overwelmingly viewed with distrust; As one in-married woman stated, “We don’t eat imported chicken. We eat our chicken from our houses, but we don’t eat imported chicken.” Others regarded imported food as "impure" and the cause of poor health. This was supported by dialog among mothers-in-law, one of who explained, “At the time that we were pregnant everything was pure, now it’s all Chinese and that’s why they have a problem with health.” This quote highlights that consumption of imported food and the subsequent avoidance of imported food are relatively new facets of village life, occurring within the lifetime of the mothers-in-law. Finally, this quote serves to underscore the interaction between diet and the changing agro-food system in Tajikistan. As noted with taboos during pregnancy, breastfeeding, and early childhood, the motivations of avoiding imported foods are tied up in protecting human health.

Women participants also discussed that foods with additives and foods “grown with chemicals” should be avoided by all household members, regardless of age or gender. The reason, given by one in-married woman, was that foods like sausage may have “other bad things” added during preparation. This links back to the perceptions of impurity and contamination discussed with regards to imported foods. Along these same lines, many women perceived fruits and vegetables grown with synthetic fertilizers or insecticides as unsafe. This was considered a pervasive issue in the region, where, according to one in-married women, “fruits and vegetables have a lot of fertilizer and chemicals.” The extent to which these taboos were actually practiced, however, is unclear. Fruits and vegetables were overwhelmingly perceived as healthy by participants across all FGDs. Participants simultaneously grappeled with the risk of consuming contaminated vegetables. As the evidence connecting synthetic pesticides with adverse health grows, growing methods, food choices, and dietary values may change to reflect what some participants described as "pure" food. For example, women participants reported active efforts to reduce the risks of eating contaminated fruit and vegetables through alternative growing methods. As one in-married woman explained, “Using fertilizer less, using compost instead of chemicals. The methods for combating insects… we think we will overcome these obstacles, like we will use chemicals less.”

## Discussion

Our findings point to several food taboos that restrict consumption of key staple foods and nutrient-rich fruits and vegetables for members of the Tajik household. We know from our previous work with this data that wheat, rice, and oil are the foundations the study population’s diet [17]. In Khatlon Province, approximately 73% of the average dietary energy consumption (DEC) is provided by carbohydrates, placing carbohydrate consumption in this region slightly above the national level and near the upper limits recommended by the WHO (71% and 75%, respectively) [29]. Wheat alone, in the form of breads, noodles, porridge, and dumplings (called *mantou*), accounts for more than two-thirds of total caloric intake per day [30]. That said, carbohydrates clearly represent a crucial source of daily calories for those living in Khatlon Province and Tajikistan as a whole. Fats and oils by comparison represent the second most crucial source of calories in the Tajik diet (20% of average DEC). Nevertheless, food taboos and health beliefs related to carbohydrates and fats/oils dominated discussions among in-married women, mothers-in-law, and men. Given the significance of carbohydrates, fats, and oils to the regional diet, potentially 90% of calories could be at risk should restrictive practices associated with food taboos overlap at any time. Simultaneously, food-limiting practices are informed by and respond to complex socio-ecological, economic, and politically-grounded challenges. Here we expand our analysis to consider the various ways taboos and health beliefs are embedded within such complex systems and influence community health. 

Our discussions suggest that carbohydrate and fat/oil-related taboos coincide during pregnancy and early childhood (between 6 months and 2 years of age). Antenatal food taboos call for the restriction of both oils/fats and carbohydrates to reduce the likelihood of specific pregnancy-related health hazards: morning sickness and difficult delivery, respectively. Nausea and vomiting in the first trimester of pregnancy, commonly referred to as “morning sickness,” is widely experienced by women during pregnancy. Symptoms typically peak 6–18 weeks into pregnancy and subside mid-way through the second trimester [31]. In Khatlon Province, oily and fatty foods are considered taboo during this period of pregnancy because they exacerbate morning sickness symptoms. Food aversion during pregnancy is widely documented and estimated to affect 50-90% of women globally [32]. That said, diet modification in response to morning sickness symptoms may result in inadequate intake of calories and nutrients if dietary changes compromise the consumption of local staples [33]. In the context of Khatlon, the risk of negative health outcomes brought on by food aversion is greatest during times of food scarcity when supplemental, non-taboo foods are more expensive or unavailable. Khatlon Province seasonally experiences food insecurity due to harsh winters and, increasingly, climate change-induced crop failure [34]. Supporting prenatal nutrition in the face of these challenges depends on sufficiency of supplemental food choices that do not agitate morning sickness symptoms. 

During this sensitive period of pregnancy, women also avoid carbohydrates to suppress gestational weight gain (GWG). Participants reported that greater GWG contributes labor complications associated with delivering an infant of higher birth weight. This belief, previously unstudied in Tajikistan, has been reported in rural Ethiopia, Nigeria, the Central African Republic, among other contexts [3, 35, 36]. While excessive weight gain during pregnancy can pose risks to antenatal health, moderate GWG (15–40 pounds depending on the baseline BMI of the woman) is a natural outcome of pregnant women meeting the increased energy requirements of pregnancy [37]. Our findings indicate that women in Khatlon Province may experience a reduced capacity to access and allocate sufficient food to support prenatal health and fetal development during the first 18 weeks of pregnancy due to the overlap of carbohydrate- and fat-limiting taboos. Poor nutrition during this phase in pregnancy, considered the “critical window” for the developing fetus due to rapid cell proliferation, impedes the development of organs and survival of the child [38].

While the restriction of oil and fat subsides with decreased likelihood of morning sickness, carbohydrates are considered taboo for the full duration of pregnancy. This appears connected to the nature of the health hazard being avoided: morning sickness is most relevant during the first two trimesters, while the fear of delivering a large baby is continual until pregnancy is complete. However, continued exclusion of carbohydrates from the prenatal diet can contribute to maternal undernutrition, which holds additional implications for child health as the primary cause of low birth weight (LBW; weight of under 2500 g). In Tajikistan, maternal undernutrition is considered the leading driver of the country’s high neonatal mortality rate (52 deaths per 1000 live births) [19]. Low birth weight is also associated with long-term developmental outcomes including subnormal growth, illness, and cognitive problems [39].

While the maternal and child health risks associated with LBW are considerable, the worries voiced by participants concerning GWG, birth weight, and risky delivery are well-founded. Evidence from public health research substantiates that heavier birth weight (4000 g or more) can pose serious risks for the mother and child [38, 39]. The possibility of obstetric complications is even higher for mothers who experienced chronic malnutrition during childhood—a common occurrence in Khatlon Province—that can lead to small stature in adulthood. Smaller placenta, uterus, and narrower pelvis accompany smaller body composition and increase the possibility of uterine rupture, obstructed labor, and other serious problems [40]. Khatlon Province (and Tajikistan as a whole) has a long history of childhood stunting which, in the last decade, has gradually declined [40, 41]. Thus, food taboos that restrict the prenatal diet may have emerged to deal with obstetric complications brought on by early childhood malnutrition of mothers who, with recent improvements in nutrition, give birth to proportionally larger infants. These findings should alert practitioners of the need to address women’s concerns around risky delivery in order to influence food-limiting taboos during pregnancy. Recently, significant investment has been placed in increasing the number of deliveries assisted by a skilled provider (physician, nurse, or midwife). According to the Demographic and Health Survey, as of 2017, 95% of births are assisted by a skilled attendant nationally (over 90% in all provinces)—a significant increase from 75% coverage in 2005 [42]. Skilled birth attendants are able to respond in the case of labor complications. In light of the linkage between prenatal diet and women’s concern toward labor complications, the recent expansion of assisted delivery may play a role in reducing carbohydrate-limiting food practices during pregnancy.

In the context of morning sickness and gestational weight gain, dietary changes aimed to mitigate the negative outcomes of nausea and vomiting during pregnancy and complications during labor, respectively. Although dietary modifications respond to symptoms and concerns that are widely experienced by mothers around the world, changes in prenatal diet impact women's and children's health in context-specific ways. In the case of Tajikistan, carbohydrates and oily foods are simultaneously avoided and central to the local diet. Dietary changes around GWG stem from broader, structural inequalities that are entangled with complications during pregnancy. Upon disolution of the Former Soviet Union in 1989, newly independent Central Asian Republics faced rapid degredation of social services, growth of unemployment, and transformation of agricultural sector and, regionally, nutrition status deteriorated [43]. Regional differences in food security reflect the uneven experiences of Tajik communities in the aftermath of the Soviet crash [8]. Today, young mothers of rural Khatlon Province, who were young children at the time of Tajik independence, are situated at a generational turning point such that the nutrition status of their children will be markedly improved compared to their own at birth and early childhood. Interestingly, our participants' concerns about heavier birth weight and labor complications are echoed in other global contexts where a generational divide in nutrition status between mothers and their children is striking [3, 35, 36]. 

Women participants reported that some maternal food avoidances are supported by recommendations from local health care providers. In-married women and mothers-in-law discussed the role of physician recommendations in their interpretation of the appropriate prenatal diet as carbohydrate-limiting. It is unclear whether a miscommunication occurred as the result of patients misunderstanding medical advice, poor communication or inappropriate messaging around antenatal diets on the part of clinics and physicians, or the effective communication of poor medical advice on the part of health care providers. Due to the recent increase in skilled antenatal care coverage in Khatlon Province (87% in 2017 compared to 65% in 2005), the link between prenatal dietary recommendations and carbohydrate restriction may indicate this is a recent phenomenon[41–43]. However, given the observation of similar taboos across diverse contexts throughout the world [23] and the extent of awareness and practice of this taboo by men and women participants across different villages in the region, it is more likely that misinterpretation of medical advice reinforces long-standing taboos or that long-standing taboos confirmed the communities' subsequent interpretations of medical advice. Based on these findings, additional clarity is needed to determine the role of health care advice in carbohydrate-restricting taboos during pregnancy.

Like pregnant women in the first and second trimesters, children between 6 months and 2 years of age experience overlapping food taboos relating to staple carbohydrates, oils, and fats. Additionally, early childhood taboos encompass certain fruits and vegetables and eggs. Such taboos may restrict the diversity of foods consumed upon introducing complementary foods (consumed alongside breastmilk starting at 6 months of age) or transitioning to a solid food diet (generally after 12–18 months). These taboos are aimed to protect children’s health by lessening the risk of upset stomach and foodborne illness associated with heavy foods and certain fruits and vegetables, respectively. According to participants, cucumbers, watermelons, grapes, and tomatoes are contaminated through growing conditions in irrigated greenhouses. Food taboos related to greenhouse-grown fruits and vegetables may be indicative of broader issues relating to water, sanitation, and hygiene (WASH) as irrigation water can be a potential source of foodborne pathogens [44]. If community experiences of food and water contamination are driving early childhood food taboos, WASH research and interventions could represent an entry point into child nutrition outcomes.

Egg-related taboos among children under two appear to be preserved by mothers-in-law—who provided detailed explanation on the topic. Awareness of egg-related taboos was also observed by men’s groups, though their justification was not congruent with discussions with mothers-in-law. Mothers-in-law associated eggs with late language acquisition and gastric irritation. According to the literature, however, child egg consumption has a significant positive influence on child growth and development. Both an observational study and a randomized control trial have linked early introduction of eggs during complementary feeding to lower rates of child stunting [44, 45]. Interestingly, the random control trial also found an association between acute diarrhea and egg consumption; though it was unclear whether foodborne illness, allergy, or reporting bias contributed to that finding [45]. Given the overall potential shown by recent studies for eggs to improve child nutrition, minimizing the effect of taboos in egg consumption during early childhood may present an avenue for addressing malnutrition in Khatlon Province.

Participants reported no food taboos specific to women after giving birth. At this point in the life cycle, health beliefs around breastfeeding emerge regarding appropriate methods for lactation management. A common challenge reported in women’s FGDs was insufficient breastmilk production, the solution to which was early cessation of exclusive breastfeeding and introduction of breastmilk replacements. Several studies echo that mothers’ concerns about insufficient lactation are a key driver of early cessation of exclusive breastfeeding [46, 47]. However, as maternal milk production is tuned to infant consumption, frequent nursing is essential for maintaining milk production [46]. In this case, early introduction of complementary foods may be exacerbating insufficient lactation described by study participants. Furthermore, the introduction of solid foods or liquids (including water) before the age of 6 months increases the risk of foodborne illness among infants and negative health outcomes into childhood [48]. Health beliefs regarding the safety and nutritional benefit of feeding animal milk versus formula as a breastmilk replacement also emerged during women’s FGDs wherein animal milk was sometimes preferred. Delayed introduction of animal milk after 1 year of age is recommended for avoiding associated risks including foodborne illness, dehydration, undernutrition, development of milk allergy, and development of type 1 diabetes mellitus [49].

Previous research suggests participants’ concerns about insufficient breastmilk may be fueled by lack of knowledge and confidence around breastfeeding or limited access to information on breastfeeding [50]. In the context of Tajikistan, knowledge of appropriate breastfeeding practices among mothers-in-law is another likely determinant of breastfeeding practices. This is supported by the literature, which suggests that senior women play a central role in determining initiation and duration of exclusive breastfeeding. Their impact can be supportive, providing young mothers with valuable knowledge and experience, or negative should they lack accurate knowledge about appropriate feeding practices [51]. Given the hierarchical household relationships observed in Khatlon Province, wherein senior mothers-in-law are respected by junior in-married women, ensuring 6 months of exclusive breastfeeding will require tapping into those structures to encourage positive, supportive relationships and a strong knowledge base across both senior and junior women.

Overwhelmingly, our findings suggest that food taboos and health beliefs disproportionately affect those whose intersecting identities confer greater nutritional vulnerability within household hierarchies at specific points in the life cycle. While taboos relating to pregnant women and young children were pervasive in the data, somefood taboos were unspecific to any subpopulation within the communities. Interestingly, unspecific taboos only emerged in our discussions with women, suggesting that women are the holders, managers, and preservers of this knowledge [18]. According to women participants, imported and processed foods and fruits and vegetables grown under certain conditions should be avoided because of the possibility of contamination by agro-chemicals that could lead to poor health among any consumer, regardless of age or gender. Unspecific taboos are indicative of women's changing roles in agriculture. Though women have been involved in agriculture throughout Tajik history in managing kitchen gardens, the fall of the Soviet Union catalyzed women’s entry into larger scale, waged food production when a sudden drop in employment triggered the rise in men migrating for work [26]. In the absence of men, women filled many traditionally masculine occupations, agriculture among them. Today, 75% of women in Tajikistan are involved in waged agricultural labor [16]. A second consequence of the fall of the Soviet bloc was the sudden drop in agricultural inputs entering former Soviet bloc countries, which plummeted to less than one-third of their former value within 3 years [52]. Food systems changed, naturally, in the hands of women. Women held generations expertise in low-input growing methods and received limited access to agricultural inputs (e.g. fertilizers, pesticides, improved seeds, high-quality irrigation, extension services) traditionally targeted towards men in agriculture [53, 54]. Thus, implementation of low-input agriculture accompanied women’s entry into larger-scale farming as a result of necessity and familiarity [26].

Cultural values are responsive to behavior, and vice versa. This relationship may be heightened in the face of extreme consequences. In the case of post-Soviet Tajikistan, the threat of starvation facing citizens the mid-1990s *demanded* changes in values and practices throughout the food system. The emergence of unspecific food taboos may represent a response to emerging values around low-input, domestically produced food products. Interestingly, similar trends have been reported in other countries that shared close economic ties to Soviet Russia and experienced extreme challenges to food security after 1991, most prominently Cuba [52].

The gendered terrain of production and reproduction in Tajikistan is dynamic and fluid. Men’s transience amidst waves of out-migration confers instability to their identities and traditional roles while women occupy new, formerly masculine spaces within and outside of the home. It is unclear how new household relations play out in the absence of men. Our findings from focus groups and participant observations suggest the continued dominance of the mother-in-law as the informal head of house. The experience of in-married women is likely to depend heavily on her relationship with her mother-in-law [17] facilitate. Additionally, the knowledge and confidence displayed by in-married women and mothers-in-law during FGDs brought to light clear gender differences in health and nutrition knowledge between men, mothers-in-law, and in-married women. While women gave consistent responses regarding which foods were taboo and why, men were often unable to provide detailed or congruent information. As suggested in previous studies, such health and nutrition knowledge may be preserved by women, who pass knowledge are related practices from mother to daughter and from mothers-in-law to daughters-in-law [15]. Women were also comparatively more active during FGDs, engaging and debating with fellow participants, while men were more hesitant in their contributions. This may reflect lack of confidence among men to contribute discussions situated beyond familiar terrains of knowledge. However, differentiation along gendered knolwedges may be perpetuated by long-standing stereotypes that classify nutrition as a “women’s issue” (separate from masculinity) and include women (while excluding men) in nutrition interventions [4]. The focus on women in the health sector is blatant within large survey datasets, which house rich information on the Tajik context while nearly excluding men’s health statistics (For example, [41]). Researchers across gender and health increasingly emphasize that gender hegemonies operate through *both* masculinities and femininities and, in this way, are mutually reinforcing [4]. The impact of such gender orders is further compounded according to the co-experiences of age, race, class, education status, caste, among other identities. Furthermore, migration of men out of Tajikistan is a destabilizing force that can affect household nutrition [26]. Thus, while women and men may face unique health priorities attached to their position within the broader socioecological context of Tajikistan, women’s and men’s health are inseparable [9].

Interventions that address the gendered nature of health knowledge and the dynamic intrahousehold arrangements unique to Tajikistan require practitioners to actively engage with all members of the family. Nutrition interventions that engage men and women can address the broader sociocultural factors that influence food taboos and health beliefs. A recently published review showed that men’s involvement in carefully planned health interventions can improve men’s knowledge of good household nutrition practices [55]. Additionally, the study revealed that men who are engaged in household nutrition interventions can encourage adoption of supportive health knowledge and behaviors by other household members. Lastly, involvement of men and women together in nutrition interventions can contribute positive changes in marital relationships. In the context of Tajikistan, engaging men may serve to emotionally empower men as fathers and as decision-makers regarding their own health status by narrowing the gender gap in health literacy and minimizing men’s isolation from the family. This is particularly relevant to Tajik families that experience frequent or son for work/or son for work [55].

This research represents the necessary first step toward building an understanding of the potential impact of food taboos and health beliefs on household nutrition in Tajikistan. However, several limitations must be considered when interpreting these findings. First, health beliefs and food taboos may be associated with geographic proximity to nutrition and health services and vary by the participant’s education status. The villages were selected based on participation in the Women’s Economic Empowerment Program (WEEP) activities, therefore FGDs were arranged to accommodate WEEP members who may have more in-depth knowledge of appropriate health and nutrition practices due to their involvement in the program. Also, this may contribute to the stark difference in health literacy demonstrated by men and women. Finally, researchers faced difficulty reaching saturation within men’s FGDs due to the high rate of male migration. In some villages, men who met inclusion criteria for this study were completely absent. Therefore, the lack of men in these villages reflects the small sample size among this target population. Furthermore, as this study represents formative work on the intersection of food taboos and nutrition in Tajikistan, continued research is necessary to further characterize and define the nuances within this area of study. For example, investigations into nutrition in Khatlon call for additional study around the extent to which food taboos are practiced and their impact on nutrition status via collection of anthropometric data. Rich ethnographic data would further illuminate the recent interactions between migration, gender, and health.

Despite considerable investments in nutrition education in the last 30 years, little progress has been made in identifying interventions that contribute to sustained, long-lasting improvements. The unclear outcomes of these programs may reflect the limited attention placed on addressing social norms, cultural practices, and historic factors that contribute to dietary practices. This research contributes to that effort in Tajikistan by identifying food taboos and health beliefs that may impact nutrition and characterizing them within the sociocultural context of Khatlon Province. Our study suggests that gender plays a significant role in shaping dietary knowledge and practices in the study population. Similar connections between gender and knowledge are reflected in the findings of other scholarly works (See [18]). Analysis of gender-differentiated dietary practices and knowledges serves to illuminate intersecting patterns of social difference that contribute to various health outcomes by moving beyond investigation along a man-woman binary. A gender relations approach looks closely at differentiated categories within gendered groups and their relations therein, such that food practices and nutrition are conceptualized within the contexts of power, history, environment, economics, and politics in which they are embedded [9]. We hope the findings of this study are supportive for guiding nutrition-sensitive extension work that engages *all members* of the household within efforts to improve nutrition. Extension programs that seek to integrate these findings into future work should (1) address the sociocultural arrangements that perpetuate food taboos among vulnerable members of society; (2) focus on critical points in the life-cycle nutrition status which is most vulnerable; (3) consider labor migration as a destabilizing factor within men’s, women’s, and children’s’ health, and (4) address emerging unspecific food taboos and health beliefs as they relate to changing values and cultural beliefs within Tajikistan.

## Conclusion

In exploring the gender dynamics of nutrition, the interactions between local knowledge and the evolving political, economic, and environmental context of Khatlon Province, Tajikistan comes forward as central to local adaptive strategies around health. Food taboos and health beliefs are situated within and shaped by these integrated processes and thus cannot be divorced from them. This study details how these embedded interactions can influence health outcomes like nutrition status. Gender and age emerged as intersecting subjectivities that reproduce hierarchical familial arrangements while holding implications within and beyond the household. As seen in other contexts, the social interactions through which performance of gendered subjectivities takes place are saturated with power [55, 56]. Our study further explored the role of intrahousehold relations in reproducing gendered knolwedge and practices around health and diet; The focus on food taboos and health beliefs reflect themes identified during previous research (see [17]). We found differential implications of adaptive health practices and beliefs among the subpopulations identified during focus ground discussions. Vulnerability (here, defined as a comparatively higher susceptibility to malnutrition, severity of its effects, and risk of long-term health consequences due to poor nutrition) was concentrated among those whose intersecting subjectivities conveyed a lower position within the household social structure at specific points in the life cycle. These patterns may contribute immediate health impacts among in-married women and children under the age of two. Among these subpopulations, increased physiological needs intersect with restricted intake of carbohydrates, the foundation of many staple Tajik dishes. Based on participant discussions, we present food taboos as dynamic, flexible, and in a constant state of emergence in response to ongoing socioecological changes; the topics of shifting demographics, agricultural labor, and unspecific taboos were most salient in this respect. While men did not practice any food restrictions, the instability of migration inherent to their transience in family and community life may convey novel challenges to their health. However, men's health is globally understudied and men's presence as actors in nutrition-sensitive initiatives is minimal—save as a comparative model by which to measure the status of women. Recognizing the mutual constitution of health across gendered subjectivities is crucial to long-term improvements in population wellbeing. 

According to the findings of this study, an increase in agricultural production is insufficient to improving household nutrition status. Instead, it is crucial for organizations to rethink the way nutrition-sensitive interventions are planned and implemented. While targeted appraoches to malnutrition can hold value, they can also impose unintended consequences when behaviors and beliefs are extracted from their location within a dynamic socialenvironment complex. Among the opportunities for change, a gender relations approach to understanding health can transform the systems that separate gendered experiences into silos. This study is situated within the context of agriculture extension services due to their potential to pursue plural strategies to improved health where agriculture is the dominant livelihood. Agriculture extensionists hold a unique position at the nexus of agro-food systems, nutrition, and gender and are able to build meaningful participant-led interventions through long-term relationships with communities. Such involvement at the local level is necessary for nuanced practice-based work within complex processes described in this article. This research has applications beyond extension and agriculture sectors, however, and we call on scholars and practitioners of diverse epistemologies to draw connections to their many areas of inquiry. 

## Abbreviations

AES: Agriculture extension services
DEC: Dietary energy consumption
EHE: Extension home economist
FGD: Focus group discussion
GWG: Gestational weight gain
IPM: Integrated pest management
IYCF: Infant and young child feeding
KII: Key informant interview
LBW: Low birth weight
TAU: Tajikistan Agrarian University
TAWA: Tajikistan Agriculture and Water Activity
UF: University of Florida
USAID: United States Agency for International Development
WEEP: Women Entrepreneurship for Empowerment Project
